# Deep Learning-Based Recognition of Cervical Squamous Interepithelial Lesions

**DOI:** 10.3390/diagnostics13101720

**Published:** 2023-05-12

**Authors:** Huimin An, Liya Ding, Mengyuan Ma, Aihua Huang, Yi Gan, Danli Sheng, Zhinong Jiang, Xin Zhang

**Affiliations:** 1 Department of Pathology, Sir Run Run Shaw Hospital, Zhejiang University School of Medicine, Hangzhou 310016, China; 2 Zhejiang Dahua Technology Co., Ltd., Hangzhou 310053, China

**Keywords:** high-grade squamous intraepithelial lesion, p16, diagnosis, artificial intelligence, deep learning

## Abstract

Cervical squamous intraepithelial lesions (SILs) are precursor lesions of cervical cancer, and their accurate diagnosis enables patients to be treated before malignancy manifests. However, the identification of SILs is usually laborious and has low diagnostic consistency due to the high similarity of pathological SIL images. Although artificial intelligence (AI), especially deep learning algorithms, has drawn a lot of attention for its good performance in cervical cytology tasks, the use of AI for cervical histology is still in its early stages. The feature extraction, representation capabilities, and use of p16 immunohistochemistry (IHC) among existing models are inadequate. Therefore, in this study, we first designed a squamous epithelium segmentation algorithm and assigned the corresponding labels. Second, p16-positive area of IHC slides were extracted with Whole Image Net (WI-Net), followed by mapping the p16-positive area back to the H&E slides and generating a p16-positive mask for training. Finally, the p16-positive areas were inputted into Swin-B and ResNet-50 to classify the SILs. The dataset comprised 6171 patches from 111 patients; patches from 80% of the 90 patients were used for the training set. The accuracy of the Swin-B method for high-grade squamous intraepithelial lesion (HSIL) that we propose was 0.914 [0.889–0.928]. The ResNet-50 model for HSIL achieved an area under the receiver operating characteristic curve (AUC) of 0.935 [0.921–0.946] at the patch level, and the accuracy, sensitivity, and specificity were 0.845, 0.922, and 0.829, respectively. Therefore, our model can accurately identify HSIL, assisting the pathologist in solving actual diagnostic issues and even directing the follow-up treatment of patients.

## 1. Introduction

Cervical cancer is the fourth leading cause of morbidity and mortality in women worldwide, representing a major public health problem [1], while approximately 75% of cervical cancers occur in developing countries [2]. Epidemiological and molecular studies have shown that persistent human papillomavirus (HPV) infection has been identified as a leading factor in cervical cancer. The human papillomavirus deoxyribonucleic acid (DNA) test and thin-prep cytologic test (TCT) are used to screen the status of cervical lesions among all women aged 21 to 65 years [3,4]. The implementation of cervical screening programs has improved the detection rate of precancerous lesions. At the same time, the number of cervical biopsy specimens has gradually increased, increasing pathologists’ diagnostic workload. Accurate pathological diagnosis allows for the early detection of cervical precancerous lesions, which increases the chances of successful treatment and cure.

Cervical intraepithelial neoplasia (CIN) is a premalignant lesion of cervical cancer caused by persistent HPV infection, especially with the high-risk HPV subtype [5]. According to the proportion of basal-like undifferentiated cells in the epithelium, which reflects the loss of epithelial cell maturation, CINs are classified into three grades: CIN1, CIN2, and CIN3 [6]. Since 2012, the lower anogenital squamous terminology (LAST) standardization project has proposed a two-tiered nomenclature to replace the three-tiered CIN system, where condyloma and CIN1 are grouped under the low-grade squamous intraepithelial lesion (LSIL), and CIN2 and CIN3 are classified as a high-grade squamous intraepithelial lesion (HSIL) [7]. The LSIL is characterized by the proliferation of basal/parabasal-like cells in the lower third of the epithelium that may show mitotic activity, albeit usually without atypical mitoses, along with koilocytic atypia with clearly retained features of maturation/differentiation. The HSIL (CIN2) shows basal/parabasal-type atypia in the lower two-thirds of the epithelium, and the nuclear abnormality is shown to be hyperchromatic with irregular coarse chromatin membranes. The HSIL (CIN3) shows full-thickness atypia and mitotic activity extending into the full-thickness epithelium. Clinically, a high-grade squamous intraepithelial lesion is the cutoff point for cervical excision treatment to prevent further progression to cervical cancer, which affects patients’ survival, mortality, and healthcare costs [8,9]. Morphological diagnosis and grading of cervical squamous intraepithelial lesions have significant variability among different seniorities of pathologists. If there is a professional disagreement in the histologic interpretation, p16 and Ki67 immunohistochemistry (IHC) will be used to assist in the diagnosis. Co-staining of p16 and Ki67 has the potential to distinguish HSIL from benign lesions, such as basal cell hyperplasia and reactive atypia [10]. Although IHC may be helpful in limiting tissue biopsies and eliminating interobserver variability, histopathology remains the gold standard for diagnosis. Actual diagnostic work is still highly dependent on histologic morphology, and women with inaccurate HSIL diagnoses may be undertreated or overtreated. The treatment options for cervical precancerous lesions can vary depending on the severity of the lesion. Therefore, a new and intelligent diagnostic system is required to increase diagnostic accuracy and reduce the diagnostic burden.

The rapid development of artificial intelligence in digital pathology is quietly revolutionizing tumor diagnosis [11]. The current focus in digital pathology on cervical disease is primarily centered on classification and grading. For instance, Calik et al. [12] proposed two different classification schemes that utilize local histograms and cell morphometric features for tissue classification based on Kullback-Leibler divergence, which achieved an accuracy of 78.69%. Keenan et al. [13] attempted to develop an objective grading system using machine vision by analyzing the architectural features of the cervical epithelium. Their system was able to identify CIN3 in 98.7% of cases. Convolutional neural networks (CNNs) can identify cervical squamous cell carcinoma (SCC) and cervical adenocarcinoma (AC) with an AUC of 0.98 and 0.966, respectively [14]. These studies demonstrate that artificial intelligence models have good performance in the field of cervical pathology.

Currently, the automatic grading of SILs broadly falls into two categories. The first category involves quantifying features observed by pathologists, such as cellularity and nuclei, which are then used by classifiers such as support vector machines (SVM) and linear discriminant analysis (LDA) [15,16]. The second category of methods involves using CNNs for an end-to-end CIN classification, which has been shown to be more effective [17]. As SILs undergo a complex cell division and differentiation process, the boundaries of grading are difficult to define, and the end-to-end classification based on CNN is arbitrary and not suitable for visual auxiliary pathological diagnosis. In addition, the high similarity of pathological cervical images and pathologists’ inter- and intra-variation can lead to misdiagnoses and missed detection of cervical precancerous lesions. In this background, our study aimed to handle the problem of insufficient feature extraction and incorporate p16 and Ki67 IHC to enhance the diagnostic accuracy of HSIL, thus helping patients be cured before cancer develops. Artificial intelligence algorithms can analyze large amounts of image data and identify patterns that may not be immediately apparent to a human observer.

Although there are numerous applications of AI in tumor pathology, the study of precancerous disorders is uncommon. Our study aimed to build an effective deep-learning algorithm for HSIL detection. We established the AI model to predict and highlight the p16-positive areas and HSIL regions using Swin-B based segmentation [18]. Furthermore, we evaluated the AI model at both the patch level and the patient level. Only the whole slide images (WSIs) of the cervical biopsy slice are needed for basic analysis. Additionally, we explored whether pathologists could improve their diagnostic performance with the assistance of the AI diagnosis model when reading cervical biopsy images. Overall, the main contribution of this study is the accurate identification of HSIL in the cervical squamous epithelium, thus resolving the problem of missed diagnosis and misdiagnosis due to the high similarity with cervical LSIL and benign proliferative lesions and ultimately guiding treatment.

## 2. Materials and Methods

### 2.1. Data Collection

The datasets for this study were collected from 111 female patients who underwent colposcopy and cervical biopsy at Sir Run Run Shaw Hospital, Zhejiang University School of Medicine (SRRSH), in 2021. Institutional review board approval was obtained for this study on 6 December 2022 (Approval No. 0471). In total, we collected 111 cases, 111 hematoxylins and eosin (H&E)-stained slides, and 197 IHC slides. Since cervical biopsy pathology is not a single lesion in most instances and the normal epithelium, LSIL region, and HSIL region are mixed with each other, it is not possible to simply diagnose a case as LSIL or HSIL. Therefore, to test the entire algorithm network on a set of independent sides, 80% of the 111 cases were used as the training set and 20% as the test set. All the slides were formalin-fixed paraffin-embedded (FFPE) sections. H&E slides and immunohistochemical slides were scanned at 40× magnification (0.25 µm pixel^−1^) by a digital pathology scanner (KF-PRO-400, KFBIO). Sensitive information such as the patient’s name, medical record number, and ID number were removed from the files.

### 2.2. Data Preparation for Model Training and Evaluation

The dataset is split randomly and stratified according to the distribution of HSIL and on-HSIL. The labeling processes for training were prepared by pathologists through annotation and image registration. The normal epithelium, LSIL, HSIL, and mitosis were annotated by pathologists. The p16-positive region was automatically labeled on the H&E slides using a pre-trained Whole Image Net (WI-Net) and image registration with the p16 IHC image.

#### 2.2.1. Dataset Division and Labeling

The ground truth was established on the basis of the original SRRSH pathology report and confirmed by two experienced pathologists (who worked for more than 10 years) to ensure an error-free diagnosis. Each H&E slide had corresponding p16 and Ki67 IHC slides. The diagnosis was made according to the H&E morphology and the expression of p16 and Ki67. The final ground truth was based on a consensus between the two pathologists. A third pathologist reviewed the slides and discussed the final diagnosis if the two pathologists had disagreements. The high-grade squamous intraepithelial lesion included CIN2 and CIN3, showing full-thickness atypia characterized by basaloid cells and mitotic activity extending into the upper two-thirds of the full epithelium, with the upper portions of the epithelium showing a significantly higher ratio of the nucleus to the cytoplasm than non-HSIL (non-HSIL refers to the normal epithelium) and LSIL. LSIL is characterized by the lower third of the epithelium demonstrating a proliferation of basal/parabasal-like cells that may show mitotic activity, showing koilocyte atypia with clearly retained features of maturation/differentiation.

In this study, the algorithm model was trained on 6171 labeled patches from 111 patients. The process generated 6171 patches, with 4921 patches from the 90 cases of the training set and 1250 patches from the 19 cases of the test set. We mainly divided the cervical squamous epithelium into two categories: HSIL and non-HSIL. In the beginning, we mainly labeled the normal epithelium as LSIL, HSIL, p16-positive regions, mitosis, etc. All annotations are mainly outlined by a junior pathologist, with one additional experienced pathologist reconfirming the annotations to ensure accuracy. The automated Slide Analysis Platform (ASAP) 1.9 software (Radboud University Medical Center, Nijmegen, The Netherlands) was used to generate corresponding masks.

#### 2.2.2. Image Registration of p16 from IHC to H&E

The annotations of the p16-positive region on the H&E slides were obtained automatically from the registration of the p16-positive region, which was detected in the p16 IHC images by a pre-trained whole image (WI)-Net [19].

**Figure 1:**
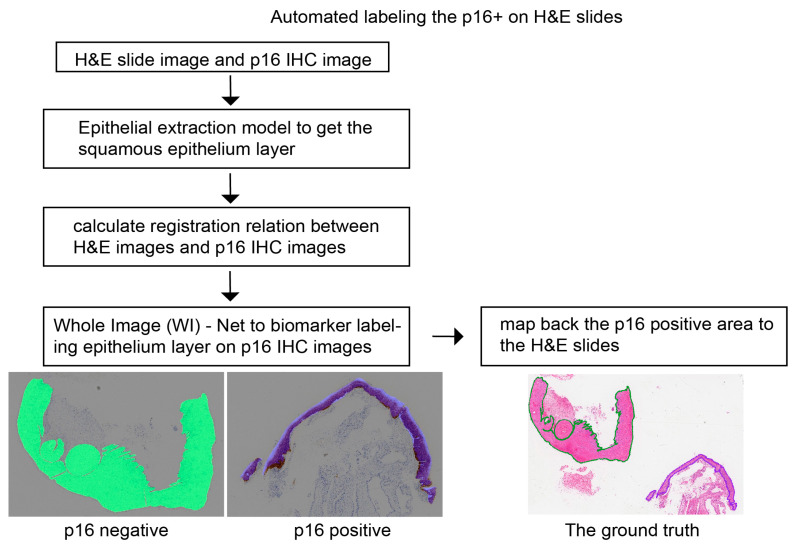
The flowchart for registering the p16-positive area in IHC to the respective H&E slide. IHC: immunohistochemistry.

Figure 1 shows the flowchart of the automated labeling of the p16-positive region on the H&E slides. We outlined the squamous epithelium layer of the H&E slides and extracted the contour ($S_{H\&E}$), which is a set of contour points:

(1) $$S_{H\&E}=\left\{\left(x_{H\&E}^{i},y_{H\&E}^{i}\right)\right\},i=1,2,\ldots ,n$$

where the $\left(x_{H\&E}^{i},y_{H\&E}^{i}\right)$ represent the position coordinates of the points, n is the number of contour points. Then, we mapped them to the p16 IHC image based on the registration relationship. The registration relation was calculated by the open-source medical image registration toolbox named Elastix [20,21], which is a two-dimensional displacement field ($\left[f_{x},f_{y}\right]$). The corresponding contour of the p16 IHC image is as follows:

(2) $$S_{IHC-P16}=\{\left(x_{H\&E}^{i}-f_{x}(x_{H\&E}^{i},y_{H\&E}^{i}\right),y_{H\&E}^{i}-f_{y}\left(x_{H\&E}^{i},y_{H\&E}^{i}\right))\},i=1,2,\ldots ,n\;$$

Next, the epithelial layer in the p16 IHC image was labeled by the pre-trained WI-Net, which is a fully convolutional network dividing cells into two groups: p16-positive and p16-negative. The last step is mapping the p16-positive area back to the H&E slides and generating the p16-positive mask for training.

### 2.3. Algorithm Development

In this work, the algorithm framework for squamous intraepithelial lesions is mainly composed of two parts: an epithelium segmentation model and a segmentation-based epithelial classification model. As is well known, CNN-based image segmentation sprang up from fully convolutional networks (FCN), transforming the classification networks into end-to-end, pixel-to-pixel architectures [22]. The Swin Transformer is a new general-purpose backbone whose representation is computed with shifted windows, limiting the self-attention within the window. Liu et al. [18] built the base Swin Transformer architecture, named Swin-B.

#### 2.3.1. Squamous Epithelial Detector and Skeleton-Based Partition

**Figure 2:**
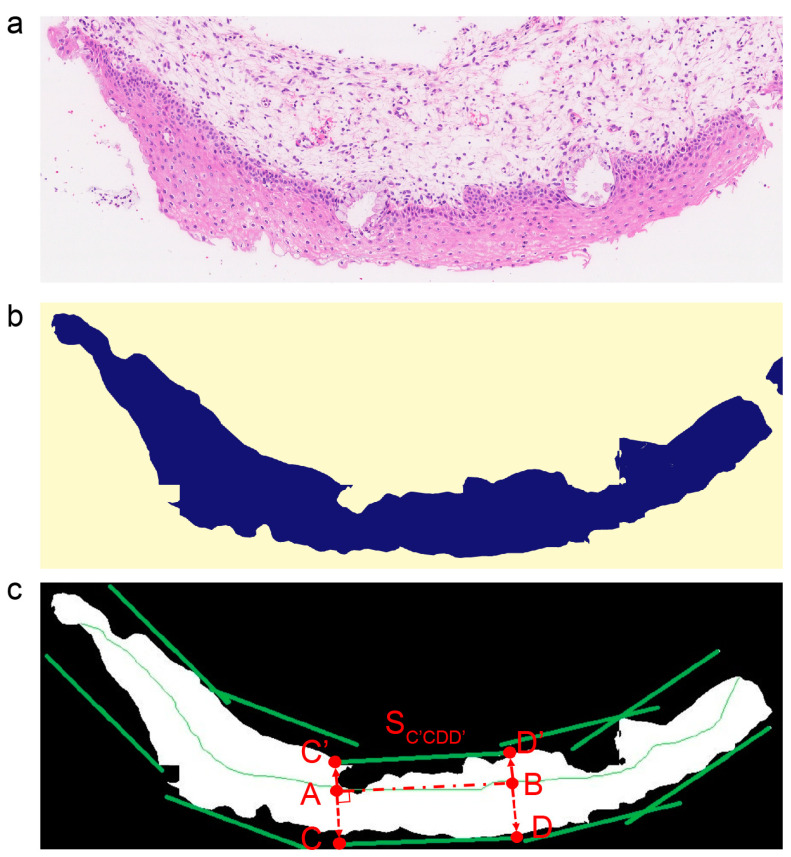
Skeleton-based partition. (**a**) The squamous epithelium layer was detected by the squamous epithelium segmentation model; (**b**) the mask of the squamous epithelium layer was segmented by the squamous epithelium segmentation model; (**c**) the schematic diagram of cutting patches based on the skeleton, where A and B are the endpoints of a bisection axis, the segment AB is translated along its vertical direction until there is no intersection with the SE mask, C (C’) and D (D’) are the endpoints of the segment obtained by translation, S_C’CDD’_ is the parted patch with the bounding box C’CDD’.

In the beginning, the squamous epithelium (SE) segmentation model based on Swin-B isolated the SE layer from the H&E slides. The WSI slides at 40 × (0.25 µm/pixel) were cut equally into fragments with a step window size of 4096 × 4096, and the fragments were scaled to a size of 512×512 as the model input. After the model inference, we obtained the binary mask of all fragments of equal size, where each pixel of the mask refers to two groups: non-SE area (0) and SE area (1). Then, all the masks were integrated back into the original slide structure, thus obtaining the mask of the whole slide. By extracting the contours of the SE area (1) on the mask, we got the corresponding bounding boxes of each SE layer and cut them out, as shown in Figure 2a,b.

Then, in order to keep the growth orientation of the epithelium in image patches for tissue-level analysis, skeleton-based epithelium partition [15,23] was used to obtain vertically divided patches. The skeleton medial axis of each epithelium was extracted based on the distance transform, and the shorter axis was cut off. Additionally, the longest axis was divided equally into the bisection axis, with a length of 4096. As shown in Figure 2c, A and B are the endpoints of the bisection axis, and AC and AC’ are the vertical direction segments of the line segment (AB). The line segment AB expands rapidly along AC and its opposite direction, AC’, until there is no intersection with the SE mask. After that, two line segments, CD and C’D’, were obtained, which are the bounding line segments of the patch (S_C’CDD’_). Additionally, these patches were taken as the minimum units to provide auxiliary diagnostic indexes.

#### 2.3.2. Squamous Epithelial Tissue Analysis

**Figure 3:**
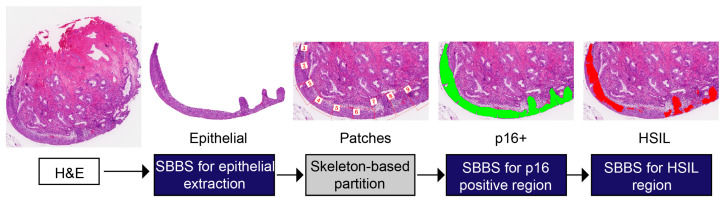
The whole slide image classification workflow. SBBS (Swin-B based segmentation).

The whole slide image classification workflow is depicted in Figure 3. After the epithelial patches were extracted, we carried out the squamous epithelial tissue analysis. In the diagnosis of the squamous epithelium, the results of immunohistochemistry have a significant auxiliary effect for pathologists. If it is possible to predict areas on H&E slices where immunohistochemistry may be positive, then this can force pathologists to pay attention to these key areas. To this end, we used the Swin-B-based segmentation net to mark the potential p16-positive area on the patches. Thus, we got the binary mask from the segmentation, referring to two groups: the p16-negative area (0) and the p16-positive area (1). The model was trained with cross-entropy loss and dice loss. The formula is as follows:

(3) $$\mathrm{the}\;\mathrm{CELoss}=-\overset{512}{\underset{j=1}{\sum }}\overset{512}{\underset{i=1}{\sum }}y_{i,j}\log\left(\rho_{i,j}\right)$$

(4) $$\mathrm{DiceLoss}=1-\frac{2\times TP+\varepsilon }{FP+2\times TP+FN+\varepsilon }\;$$

where $\rho_{i,j}$ is the probability of the pixel (i,j) prediction being positive (respectively representing the SE, p16-positive, and HSIL among different models); $y_{i,j}$ is the ground truth label, which is either 0 or 1; *TP*, *FP*, and *FN* represent the pixel numbers of true positive, false positive, and false negative, respectively; ε is the smoothing parameter, set as 1.

In the next step, we carried out an HSIL diagnosis on the p16-positive patches. Following the common approach in computational pathology, we constructed a CNN classification model to classify the p16-positive patches into two classes: non-HSIL (0) and HSIL (1). The classification model was based on the ResNet-50 backbone, whose parameters were pre-trained with the ImageNet dataset, and the model input size was set to 512×512. In the prediction phase, patches were divided into classes with the highest prediction probability. To enhance the interpretability of AI model analysis, we used segmentation technology to transform the direct diagnosis into the segmentation of the HSIL area. The model used the same model architecture and hyper-parameters as the p16 segmentation model. After obtaining the binary mask ($S_{HSIL}$) for each pixel referring to two groups: non-HSIL (0)/HSIL (1), we calculated the HSIL diffuse proportion ($DiffuseP_{HSIL}$), and in our research, patches were predicted to be HSIL when their $DiffuseP_{HSIL}$ was above the threshold of 10%. The formula is as follows, where $S_{SE}$ is the SE binary mask of the patches:

(5) $$the\;DiffuseP_{HSIL}=\frac{\sum S_{HSIL}}{\sum S_{SE}}\;$$

It should be noted that there were only 961 HSIL patches among all 4921 patches in the training set; therefore, we adopted a balanced sampler to get each batch during the training phase, and the cross-entropy loss was weighted according to the proportion of the class sample quantity. The formula is as follows:

(6) $$\mathrm{the}\;\mathrm{CELoss}=-\overset{512}{\underset{j=1}{\sum }}\overset{512}{\underset{i=1}{\sum }}(1-w_{c})y_{i,j}\log\left(\rho_{i,j}\right)\;$$

where $w_{c}$ is the sample proportion of class (c).

#### 2.3.3. Model Training

Our AI models were all built using the OpenMMLab series (https://github.com/open-mmlab accessed on 5 May 2022), which is the most complete open-source algorithm system designed for different directions of computer vision, such as MMClassification for image classification, MMDetection for object detection, and MMSegmentation for semantic segmentation. All experiments were conducted using the Ubuntu 18.04 system with 8 NVIDIA GPUs (GeForce GTX 1080 Ti) for multi-GPU training. Additionally, the MMSegmentation version we used was 0.25.0, and the MMClassification version we used was 0.19.0. The software we used included CUDA 10.2 and cuDNN 7.6.5 for GPU acceleration, PyTorch 1.6.0 and Torchvision 0.7.0 for model construction and training, OpenCV 4.4.0 for image processing, especially in the skeleton extraction step, and OpenMP 4.5 for multi-GPU training. We applied image augmentation during the training process, such as RandomRotate, Flip, Blur, RandomBrightnessContrast, and HueSaturationValue. The input size of the AI models was set to 512×512.

All the segmentation models were constructed by means of the MMSegmentation toolbox; the backbone of the segmentation model is Swin-Base (Swin-B), the decode head is UPerHead (the decode head of the UPer-Net Unified Perceptual Parsing Network), and FCNHead is used as the auxiliary head. The AdamW (Adam + weight decay) optimizer was employed to update the model with the following settings: learning rate: 6 × 10^−5^; betas: 0.9, 0.999; weight_decay: 0.01. We preserved the model weights when there was no improvement after consecutive epochs of training.

### 2.4. Evaluation of the Clinical Impact of the AI Workflow and Performance of Pathologists

In order to evaluate the effectiveness of the current AI-assisted pathological workflow and the proficiency of pathologists, a total of 19 WSIs of the cervical biopsy were utilized for testing. These digital images were subjected to deep learning algorithms to detect cervical squamous epithelium with pathological variations and assign appropriate labels. Four pathologists were recruited: two junior pathologists with less than five years of experience and two senior pathologists with at least ten years of experience. The initial diagnosis, which was based on the original pathology report from the SRRSH, was considered the ground truth. Each pathologist reviewed all 19 slides twice, once in regular (R) mode with only WSIs and once in AI-assisted (A) mode with the model segmentation and classification results. The p16 segmentation, HSIL segmentation, mitotic result, and conclusion of the AI model were provided to the pathologists to make a second diagnosis. All slides were evaluated in a random sequence, initially in R mode, followed by A mode. In order to minimize human error, the two trials were carried out one month apart.

### 2.5. Statistical Analysis

We used several different metrics to assess the performance of the model. The specificity, sensitivity, positive predictive value (PPV), negative predictive value (NPV), F1-score, accuracy, receiver operating characteristic (ROC), and area under the curve (AUC) were calculated using the Numpy, Scikit-learn, and Matplotlib packages. The two-sided P values and confidence intervals (CIs) of the AUCs used in the HSIL identification models were determined using the Delong method [24]. All the metrics above were calculated as follows:

True negative (TN): the number of patches/cases correctly identified as p16− or non-HSIL

False negative (FN): the number of patches/cases incorrectly identified as p16− or non-HSIL

True positive (TP): the number of patches/cases correctly identified as p16+ or HSIL

False positive (FP): the number of patches/cases incorrectly identified as p16+ or HSIL

Specificity: The specificity of a test is its ability to determine the p16− or non-HSIL patches/cases correctly. To estimate it, the proportion of true negatives in those cases is calculated. Mathematically, this can be stated as:

(7) $$\mathrm{Specificity}=\frac{\mathrm{TN}}{\mathrm{TN}+\mathrm{FP}}\;$$

Recall/sensitivity: The sensitivity of a test is its ability to determine the p16+ or HSIL patches/cases correctly. To estimate it, the proportion of true positives in those cases is calculated. Mathematically, this can be stated as:

(8) $$\mathrm{Recall}=\mathrm{Sensitivity}=\frac{\mathrm{TP}}{\mathrm{TP}+\mathrm{FN}}\;$$

Positive predictive value (PPV): The PPV of a test is its ability to determine the proportion of true p16+ or HSIL in the total number of positive patches/cases tested. Mathematically, this can be stated as:

(9) $$\mathrm{PPV}=\frac{\mathrm{TP}}{\mathrm{TP}+\mathrm{FP}}\;$$

Negative predictive value (NPV): The NPV of a test is its ability to determine the proportion of true p16− or non-HSIL in the total number of negative patches/cases tested. Mathematically, this can be stated as:

(10) $$\mathrm{NPV}=\frac{\mathrm{TN}}{\mathrm{TN}+\mathrm{FN}}\;$$

F1-score: the F1-score is a measure of the accuracy of the binary classification model and can be seen as a reconciled average of the model accuracy and recall

(11) $$F1-\mathrm{core}=\frac{2\times \mathrm{recall}\times \mathrm{precision}}{\mathrm{recall}+\mathrm{precision}}\;$$

Accuracy: the accuracy of a test is its ability to differentiate the p16− and p16+ (HSIL and non-HSIL) cases correctly. To estimate the accuracy of a test, the proportion of true positives and true negatives in all evaluated cases is calculated. Mathematically, this can be stated as:

(12) $$\mathrm{Accuracy}=\frac{\mathrm{TP}+\mathrm{TN}}{\mathrm{TP}+\mathrm{TN}+\mathrm{FP}+\mathrm{FN}}\;$$

## 3. Results

In this section, the experimental results of the segmentation and the classification models of HSIL are mainly shown, as are the experimental results of the segmentation models for p16 and whole slices, respectively.

### 3.1. AI-Assisted Squamous Intraepithelial Lesion (SIL) Assessment Workflow

**Figure 4:**
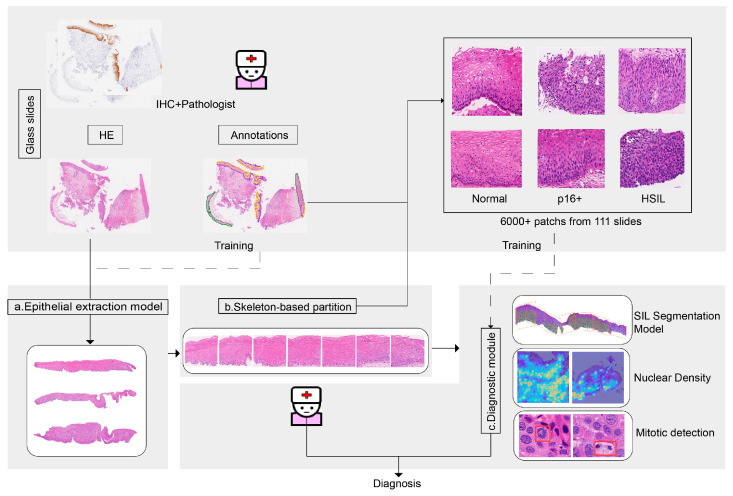
Two typical steps are involved in artificial intelligence approaches: deep learning and hand-crafted feature engineering. (**a**) Epithelial extraction model to isolate the squamous epithelium layer from the H&E slides; (**b**) Skeleton-based partition to get vertically divided patches based on a distance transform-based medial axis; (**c**) Diagnostic module: tissue-level analysis (HSIL and p16- positive area segmentation model on H&E) and cell-level analysis (nuclear density, mitotic detection).

Digital pathology now refers to AI-based digital image detection, segmentation, diagnosis, and analysis. The workflow interface is displayed in Figure 4. Upon the import of a WSI, the squamous epithelial detector is triggered to outline the squamous epithelial layers, after which the squamous epithelial layers are parted into patches. Then, the p16-positive and HSIL identification module classifies each patch as normal, p16-positive, or HSIL and highlights the lesion area. To assist pathologists with HSIL assessment, the mitotic cell is detected and highlighted.

### 3.2. p16-Positive Area Highlights and Analysis

**Figure 5:**
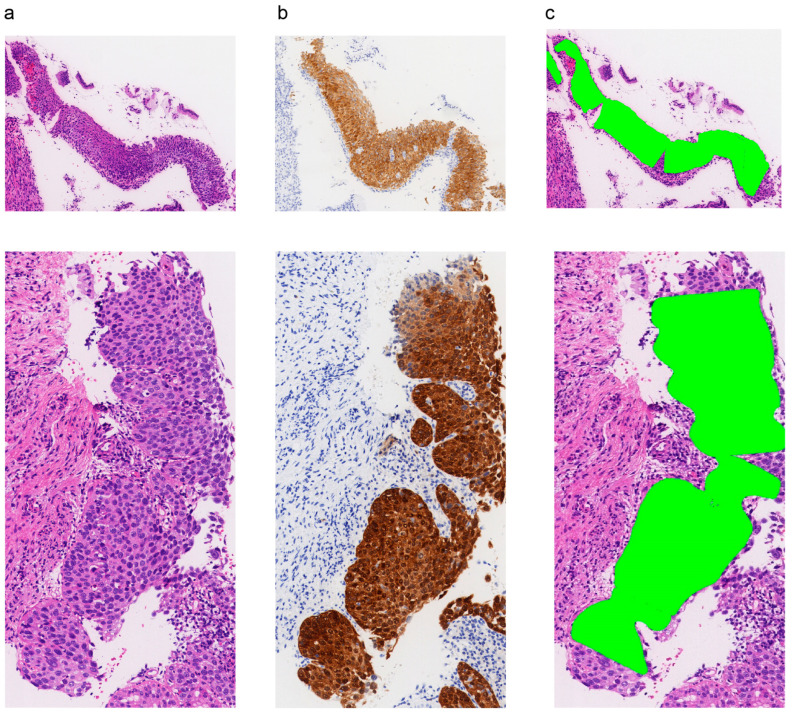
The p16-positive area highlight and analysis. (**a**) H&E of the squamous epithelium of the uterine cervix; (**b**) p16 IHC of the squamous epithelium of the uterine cervix; (**c**) the artificial intelligence model identified the p16-positive area on H&E.

The model highlighted the p16 positive areas of the H&E WSIs. In the quantitative analysis, the saliency maps generated by the algorithm achieved an intersection over union (IoU) of 72.64 on the test set. As displayed in Figure 5, the model achieved high correspondence between the predicted and actual p16-positive areas. In our research, when the p16-positive diffuse fraction was above the threshold of 5%, the patches were predicted to be p16-positive. Table 1 shows the performances of the p16 prediction models, with an average sensitivity of 0.890 [0.871–0.919] and an accuracy of 0.894 [0.874–0.921].

### 3.3. HSIL Area Highlights and Analysis

**Figure 6:**
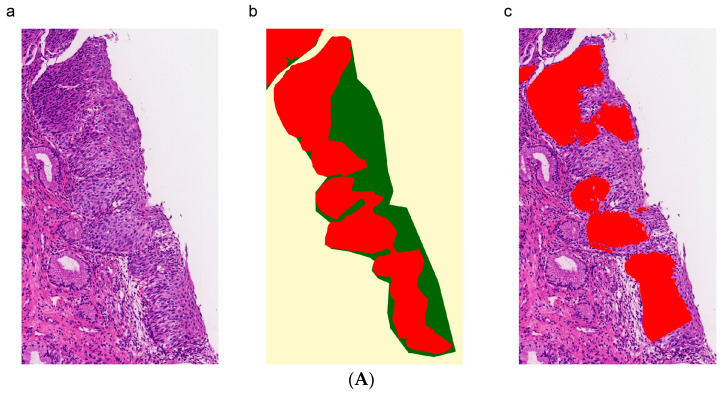

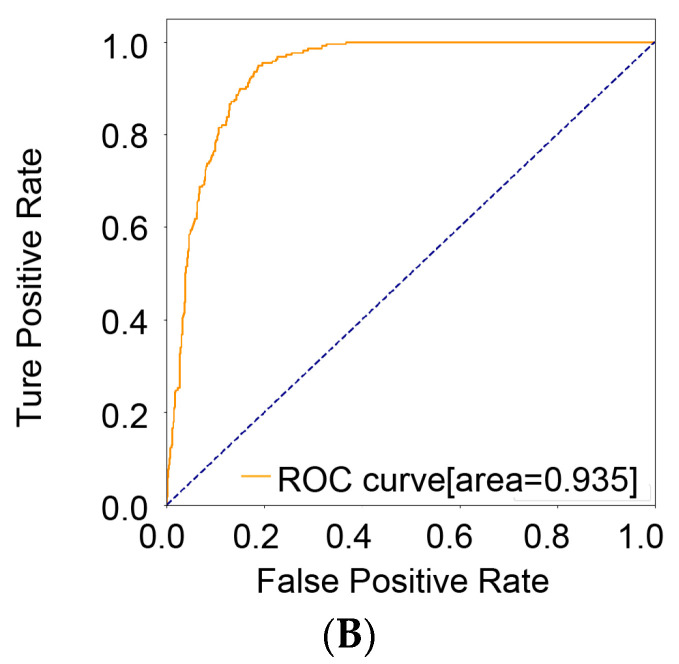
(**A**) The segmentation model can accurately identify HSIL among p16 positive areas. (**a**) H&E of the squamous epithelium of the uterine cervix; (**b**) labeled high-grade squamous intraepithelial neoplasia; (**c**) areas of high-grade squamous intraepithelial lesions detected by segmentation with Swin-B. (**B**) HSIL ROC curves for the ResNet-50 model. The area under the curve (AUC) for HSIL was 0.94 for ResNet-50.

In order to evaluate the HSIL classification performance on patches, we compared the HSIL region segmentation network with the image classification model based on the ResNet-50 backbone. For the segmentation phase, when the HSIL diffuse proportion of patches was greater than the threshold of 10%, those patches were predicted to be HSIL. Swin-B based segmentation achieved 0.914 [0.889–0.928] accuracy in the testing set for HSIL classification on patches, whereas the ResNet-50 model achieved 0.845 [0.822–0.863] accuracy (Table 2). As the HSIL region annotation was sparse, the predictions of the HSIL segmentation were more prudent but accurate. Figure 6A shows the pathologist-labeled H&E images of HSIL and the AI-predicted images of HSIL. The region of interest of the segmentation model agreed with that of the pathologist. The model successfully detected HSIL regions that were characterized by hyperchromatic atypical cells with a high nucleus-to-cytoplasmic ratio and high mitotic activity. Additionally, Figure 6B depicts the ROC curves for ResNet50, where the mean AUC was 0.935 [0.921–0.946]. For the Swin-B based segmentation model, the accuracy and sensitivity for patient level were 84.2% and 90%, respectively (Table 3).

### 3.4. Comparisons with Pathologists and a Pilot Study of AI Assistance

**Figure 7:**
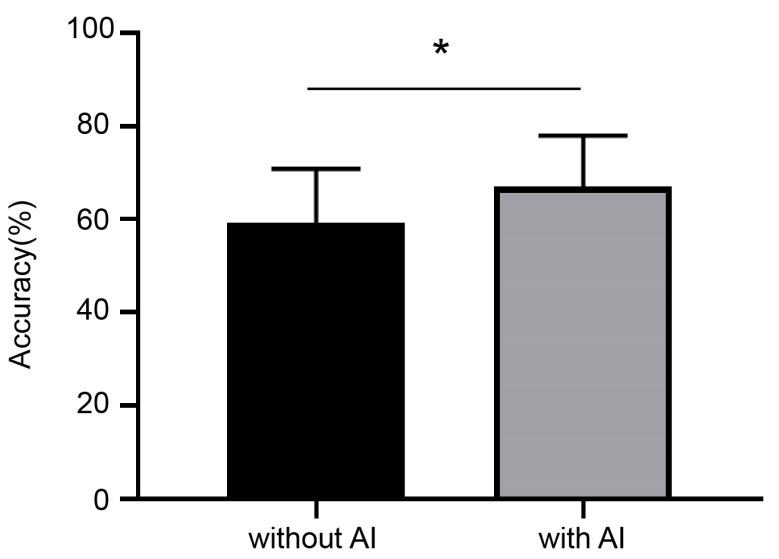
Comparisons with pathologists and a pilot study of AI assistance. The accuracy of pathologists was improved with the assistance of AI. * *p* < 0.05 (*t*-test) indicates statistically significant difference.

In order to test the AI model in clinical practice, the WSIs of the testing set were tested by four pathologists. An initial independent diagnosis was made by the four pathologists reviewing the WSIs, and a second diagnosis was obtained with the AI model. The initial diagnostic accuracy of the four pathologists was lower than that of the model. Notably, the consensus among the four pathologists was not unanimous, and some of the initial diagnoses were altered; however, all four pathologists performed better (Figure 7). The diagnostic performance of the pathologists alone versus when working with an AI model indicates that the AI model improved the pathologists’ diagnostic accuracy.

## 4. Discussion

We developed a novel AI model that achieved a high accuracy of 0.845, a high recall of 0.922, and an AUC of 0.935 (Table 2). We demonstrated that deep-learning AI can accurately distinguish HSIL regions among cervical squamous epithelium as well as p16-positive regions. Furthermore, we showed that with AI diagnostic system assistance, gynecologic pathologists can diagnose HSIL more accurately.

Extraordinary breakthroughs in artificial intelligence have been made in pathology research during the past few decades. The classification of various malignancies has been widely used to assist in diagnosis, predict prognosis, and monitor molecular changes [25,26,27]. Cervical cancer is easily preventable with early screening and diagnosis. It is acknowledged that the bulk of deep learning-based artificial intelligence has been used in cytology, colposcopy, and DNA methylation research for cervical intraepithelial lesions [28,29,30]. Jian et al. [31] showed that machine learning can identify methylation signals associated with the development of cervical cancer at qualitative and quantitative levels. Tan et al. [32] developed a CNN-based TCT cervical-cancer screening model that improved speed and accuracy and overcame the shortage of medical resources required for cervical cancer screening. One study of colposcopy conducted by Chen et al. [33] showed that AI has the potential to assist in colposcopies for the accurate diagnosis of cervical disease and early therapeutic intervention in cervical precancer. Despite the promising performance of AI with colposcopy imaging and TCT, there are still shortcomings. For example, most studies only applied static colposcopy images rather than real cervical regions to develop AI models, resulting in information bias in cervical lesion feature extraction. Furthermore, cytology-based cervical screening has poor accuracy, and the gold standard for the diagnosis of precancerous cervical lesions is histology rather than cytology. Overall, the findings suggest that AI-based deep learning algorithms have achieved better performance in all aspects of cervical cancer. Future research should focus on developing AI models that can effectively analyze histological images to improve the accuracy of cervical precancer diagnosis. The application of AI to cervical cancer screening and diagnosis has great potential to improve patient outcomes and reduce the burden of this preventable disease.

In order to effectively identify HSIL, an AI-based system was developed in this study. Different from previous studies [34] that segmented the SIL directly on the H&E image, we used Swin-B based segmentation to automatically predict the p16-positive areas in the H&E images, and we further identified the HSIL regions in the p16 areas by Swin-B based segmentation and ResNet-50 based classification. The morphological diagnosis of cervical biopsy specimens is subjective, with poor inter- and intra-observer agreement. Pathologically, the IHC of p16 has been shown to contribute to the diagnosis of HSIL/CIN2 and HSIL/CIN3, and its diagnosis is more reliable than morphology based on H&E staining alone [35]. In addition, Ki67 has been considered a sensitive biological marker of cell proliferation and CIN progression [36]. Overexpression of p16 and Ki67 correlates with the severity and progression of the cervical lesion [37,38]. The advantage of our research is that p16 prediction areas were introduced as auxiliary information to improve the diagnosis accuracy of HSIL.

The advantage of this study was that p16 prediction areas were introduced as auxiliary information to improve the accuracy of HSIL diagnosis. The results of this study suggest that the Swin-B based segmentation model achieved high correspondence between the predicted and actual p16-positive areas, indicating its potential as a reliable tool for assisting pathologists in detecting lesion regions and HPV infection. Deep learning algorithms were effective at classifying the morphological features of lesions observed in WSIs, with the segmentation model based on Swin-B achieving 91.4% accuracy for HSIL and the ResNet-50 model achieving 84.5% accuracy (Table 2). This suggests that when predicting the masks for analysis, dividing the squamous epithelium into smaller lesions contributes to improving diagnostic accuracy. It is worth mentioning that the segmentation model has been improved in most indexes to a certain degree. We speculate that small patches can better demonstrate the local features of HSIL.

Additionally, visual inspection showed that the model successfully detected HSIL regions that were characterized by hyperchromatic atypical cells with a high nucleus-to-cytoplasmic ratio and high mitotic activity. Furthermore, the ResNet-50 model generated an AUC of 0.935 [0.921–0.946] in HSIL classification (Figure 6B). These results demonstrate the potential of deep learning algorithms for detecting precancerous lesions more accurately and efficiently, with a higher clinical value. Even so, the performance of the classification model on the test set is generally acceptable; the next step should focus on improving HSIL detail differentiation. When the pathologists utilized the AI model to make a second diagnosis, they all performed better. Overall, the results are significant as they suggest that the AI model has the potential to serve as a valuable tool to assist pathologists in making more accurate and consistent diagnoses and to solve the problems of the high similarity of cervical pathological images, the inadequate experience of pathologists, and larger workloads.

Some limitations should be mentioned in this work. First, cervical intraepithelial neoplasia is a spectrum of disease progression. Different degrees of lesions, normal squamous epithelium, and stroma are mixed. Hence, it is difficult to define a clear ground truth in certain patches. Additionally, the epithelium embeds in various directions in cervical biopsy specimens, with asymmetrical forms. These factors make it difficult to extract the whole layer of the cervical epithelium. Otherwise, a rigorous histological diagnosis should consider a combination of clinical information, imaging interpretation, and fundamental histological knowledge. This makes such intricate analyses only using AI seem challenging.

In our future work, we plan to explore various avenues for improving the performance of our deep learning model in HSIL diagnosis. One potential direction is to focus on the development of multi-modal models that can leverage additional sources of information, such as patient history and clinical exam data, to enhance the accuracy of our predictions. Additionally, we will investigate methods for improving the interpretability of our model, which is critical for facilitating its adoption in clinical practice. Furthermore, we will seek to validate our model on larger and more diverse datasets, including external datasets, to establish its generalizability and robustness to variations in imaging quality and patient population. Finally, we will conduct clinical studies to evaluate the clinical utility of our model and its potential to improve patient outcomes.

The findings in this study demonstrate that the deep learning-based AI model achieves comparable accuracy to skilled pathologists in detecting HSIL and even surpasses their accuracy. Additionally, we believe that our model has great potential as an auxiliary diagnostic tool that can not only significantly improve diagnostic accuracy but also save diagnostic time and labor costs. Furthermore, the proposed deep learning-based system can help prevent misdiagnosis resulting from human error and negligence and guide follow-up treatment.

## Figures and Tables

**Table 1:** Performances of the deep learning models in predicting p16 positive regions.

Model/Class	Sensitivity (%)	Specificity (%)	Accuracy (%)	PPV (%)	NPV (%)	F1-Score (%)
Swin-B based segmentation						
p16+/p16−	89.0 [87.1–91.9]	89.6 [87.2–91.6]	89.4 [87.4–92.1]	87.0 [84.6–89.7]	91.0 [89.7–93.5]	88.0 [86.4–89.7]

**Table 2:** The HSIL performances of the CNN classification with ResNet-50 and the segmentation with Swin-B.

	Sensitivity (%)	Specificity (%)	Accuracy (%)	PPV (%)	NPV (%)	F1-Score (%)	AUC (%)
Method 1: Segmentation with Swin-B							
HSIL	64.9 [59.1–71.0]	97.0 [95.5–97.9]	91.4 [88.9–92.8]	81.0 [76.5–86.6]	93.0 [91.7–94.3]	72.0 [67.0–77.5]	NA
Method 2: CNN classification with ResNet-50							
HSIL	92.2 [89.6–95.4]	82.9 [81.4–84.7]	84.5 [82.2–86.3]	53.1 [49.7–58.2]	98.1 [97.2–98.7]	67.4 [64.6–70.8]	93.5 [92.1–94.6]

**Table 3:** The whole slide image segmentation confusion table of HSIL.

Model/Class	Sensitivity (%)	Specificity (%)	Accuracy (%)	PPV (%)	NPV (%)	F1-Score (%)
Segmentation with Swin-B						
HSIL	90.0 [85.7–94.5]	77.8 [73.4–82.5]	84.2 [80.3–90.2]	81.8 [77.5–86.4]	87.5 [83.0–93.3]	85.7 [80.6–91.6]

## Data Availability

The data used in this study is unavailable due to privacy or restrictions, and is the property of the Institute of Clinical Science, Department of Pathology, and Sir Run Run Shaw Hospital, and therefore cannot be shared.
